# Distinct properties of *Halobacterium salinarum* Agl32, an archaeal D-glucuronyl C5-epimerase involved in N-glycosylation

**DOI:** 10.1093/glycob/cwag045

**Published:** 2026-06-11

**Authors:** Antonella Aquilone, Yarin Levi, Marianna Zaretsky, Zlata Vershinin, Iris Grossman-Haham, Cristina De Castro, Jerry Eichler

**Affiliations:** Scuola Superiore Meridionale, Largo San Marcellino 10, 80138 Naples, Italy; Department of Chemical Sciences, University of Napoli Federico II, 80126 Naples, Italy; Department of Life Sciences, Ben-Gurion University of the Negev, Beersheva 84105, Israel; Department of Life Sciences, Ben-Gurion University of the Negev, Beersheva 84105, Israel; Department of Life Sciences, Ben-Gurion University of the Negev, Beersheva 84105, Israel; Department of Life Sciences, Ben-Gurion University of the Negev, Beersheva 84105, Israel; The Ilse Katz Institute for Nanoscale Science and Technology, Ben-Gurion University of the Negev, Beersheva 84105, Israel; Department of Chemical Sciences, University of Napoli Federico II, 80126 Naples, Italy; Department of Life Sciences, Ben-Gurion University of the Negev, Beersheva 84105, Israel

**Keywords:** archaea, D-glucuronyl C5-epimerase, glucuronic acid, iduronic acid, N-glycosylation

## Abstract

The N-linked tetrasaccharide decorating glycoproteins of the halophilic archaea *Halobacterium salinarum* offered the first example of N-glycosylation outside the Eukarya and still represents the only known instance of iduronic acid (IdoA) being employed in this post-translational modification. Recent identification of Agl32 as the D-glucuronyl C5-epimerase catalyzing the conversion of glucuronic acid (GlcA) into IdoA allows for comparing this enzyme in each of the three domains of life, namely, Eukarya, Bacteria and Archaea. Specifically, the current study assessed whether Agl32 requires flanking sugars on either side of the target GlcA, as do its eukaryal and bacterial counterparts. Nuclear magnetic resonance analysis of the glycan from an *Hbt. salinarum* mutant unable to add the fourth and final N-linked tetrasaccharide sugar revealed that Agl32 requires GlcA on both sides of the target GlcA for the epimerization reaction. Despite similar requirements for flanking sugars, Agl32 shares little structural similarity with eukaryal GlcE or DSepi1, D-glucuronyl C5-epimerases respectively involved in IdoA generation in the glycosaminoglycans heparin/heparan sulfate and dermatan sulfate. Moreover, Agl32 processes a substrate far shorter than what is recognized by the eukaryal enzymes. Indeed, it would appear that Agl32 relies on a catalytic mechanism distinct from that employed by these other D-glucuronyl C5-epimerases. Finally, the presence of *agl32* homologues in putative N-glycosylation gene clusters in other haloarchaea argues that the use of IdoA in N-glycosylation extends beyond *Hbt. salinarum*.

## Introduction

*Halobacterium salinarum*, halophilic archaea best known as the source of the light-driven proton pump bacteriorhodopsin (Oesterhelt and Stoeckenius 1971), provided the first example of N-glycosylation outside the Eukarya (Mescher and Strominger 1976). Subsequent analysis of the composition of the N-linked glycan decorating *Hbt. salinarum* glycoproteins revealed the presence of iduronic acid (IdoA; Wieland et al. 1986), offering the sole instance of this uronic acid being used in N-glycosylation. In Eukarya, IdoA is part of in the repeating disaccharide units comprising certain glycosaminoglycans (GAGs) such as heparin, heparan sulfate and dermatan sulfate, anionic polysaccharide chains primarily found in the extracellular matrix of animal tissues (Sarrazin et al. 2011; Casu et al. 2015; Chen et al. 2025). Eukaryal D-glucuronyl C5-epimerases responsible for converting glucuronic acid (GlcA) into IdoA have been identified (Hook et al. 1974; Maccarana et al. 2006; Pacheco et al. 2009a), as has a bacterial enzyme showing similar activity (Raedts et al. 2013). With recent identification of Agl32 as the D-glucuronyl C5-epimerase responsible for the appearance of IdoA as the third sugar of the N-linked tetrasaccharide added to selected *Hbt. salinarum* proteins (Vershinin et al. 2025), this enzyme can now be compared across evolution.

Unlike what occurs during the biogenesis of most polysaccharides, where the different sugars sequentially added to a growing chain originate as the activated monosaccharide donor, the IdoA found in GAGs is generated upon conversion of polymer-incorporated GlcA (Lindahl et al. 1972; Malmström and Fransson 1975; Valla et al. 2001). Such conversion of a polymer-incorporated sugar is a rare event, with the only other known example of epimerization of a hexuronic acid occurring at the polymer level being the conversion of β-D-mannuronic acid into α-L-guluronic acid in the biogenesis of alginates, a family of linear polysaccharides produced by brown seaweeds and certain bacteria (Haug and Larsen 1969; Valla et al. 2001). The realization that the epimerization of GlcA into IdoA occurs at the polymer level led to the discovery of GlcE (EC 5.1.3.12), the glucuronyl C5-epimerase that catalyzes this conversion during heparin and heparan sulfate biosynthesis (Hook et al. 1974). Later, DS-epimerase1 (DSepi1; EC 5.1.3.19), the glucuronyl C5-epimerase that contributes to dermatan sulfate biosynthesis, and which also acts at the polymer level, was described (Maccarana et al. 2006). DS-epimerase2 (DSepi2), a second dermatan sulfate-processing glucuronyl C5-epimerase was later discovered (Pacheco et al. 2009a). While both enzymes share a common N-terminal domain, DSepi2 contains a C-terminal O-sulfotransferase domain not found in DSepi1 (Pacheco et al. 2009a). In Bacteria, IdoA is much less prevalent than in Eukarya, having been detected in only a limited number of species as a component of a capsular polysaccharide (Lee and Cherniak 1974), the O-antigen (Hanniffy et al. 1998) and an extracellular polysaccharide (Stack et al. 1988). The only identified bacterial D-glucuronyl C5-epimerase comes from the marine bacterium *Bermanella marisrubri* sp. RED65, with its activity having been demonstrated using O-desulfated heparin as substrate (Raedts et al. 2013).

Biosynthesis of the IdoA-containing GAGs heparin and heparan sulfate begins with the assembly of a polysaccharide chain containing alternating GlcA and N-acetylglucosamine (GlcNAc) units. The GlcNAc units undergo N-deacetylation and N-sulfation, followed by D-glucuronyl C5-epimerase-mediated conversion of GlcA into IdoA, and O-sulfation of these sugars at various positions (Lindahl and Li 2009; Li 2010). The epimerase recognizes GlcA adjacent to *N*-sulfoglucosamine. Epimerization occurs more rapidly if both adjacent GlcNAc units are N-sulfated, although only sulfation of the GlcA C-4-linked sugar is essential (Bäckström et al. 1979; Jacobsson et al., 1984). In the case of dermatan sulfate, where N-acetylgalactosamine (GalNAc) alternates with IdoA in the polymer, no sugar modifications are required prior to GlcA epimerization into IdoA, with sugar sulfation occurring only after the appearance of IdoA (Malmström 1984). Details of the catalytic mechanism employed by these eukaryal D-glucuronyl C5-epimerases have come from more recent structural studies (Qin et al. 2015; Debarnot et al. 2019; Hasan et al. 2020). These efforts revealed that despite of a lack of similarity at the amino acid level, human GlcE (PDB 6HZZ) and DSepi1 (PDB 6HZN) present similarities in terms of overall shape and general organization of residues in their active sites (Debarnot et al. 2019; Hasan et al. 2020). Such analyses also provided insight into the substrate preferences of the two D-glucuronyl C5-epimerases.

With the identification of *Hbt. salinarum* Agl32 as a D-glucuronyl C5-epimerase (Vershinin et al. 2025), it is now possible to begin defining how the archaeal version of this enzyme acts, relative to GlcE and DSepi1. In *Hbt. salinarum*, the N-linked tetrasaccharide decorating glycoproteins corresponds to β-D-GlcA(2S)-(1 → 4)-α-L-IdoA(3S)-(1 → 4)-β-D-GlcA-(1 → 4)-β-D-Glc, where Glc is glucose, and S represents sulfation (Notaro et al. 2022). Agl32 acts on the GlcA found at tetrasaccharide position three in the lipid-linked N-linked glycan prior to its delivery across the membrane and transfer to selected proteins, such as archaellins, building blocks of the archaeal swimming device, the archellum (Albers and Jarrell 2012; Vershinin et al. 2025). Using a *Hbt. salinarum* glycosylation pathway mutant unable to generate this complete N-linked tetrasaccharide but rather only a precursor comprising the first three sugars, the ability of Agl32 to convert the target GlcA that now corresponds to the terminal glycan sugar was considered. In addition, the workings of the archaeal glucuronyl C5-epimerase and its eukaryal counterparts were also compared at the structural level. Finally, the possibility that other archaea involve a D-glucuronyl C5-epimerase in N-glycosylation was addressed.

## Results and Discussion

### In *Hbt. salinarum* cells lacking Agl27, the N-linked trisaccharide terminal sugar is GlcA

Eukaryal and (presumably) bacterial D-glucuronyl C5-epimerases require that a GlcA to be converted into IdoA be incorporated into a polysaccharide chain and flanked by other appropriate sugars (Hook et al. 1974; Malmström and Fransson 1975; Raedts et al. 2013). To determine whether the same holds true for the archaeal D-glucuronyl C5-epimerase Agl32, the structure of the N-linked trisaccharide added to archaellins in *Hbt. salinarum* cells deleted of *agl27* was assessed by NMR. In the absence of Agl27, corresponding to the glycosyltransferase responsible for the appearance of the fourth sugar of the N-linked tetrasaccharide normally added to target proteins (Vershinin et al. 2021), the GlcA added to position three of the N-linked glycan is now the terminal sugar. NMR analysis allows for the determination of whether or not this terminal GlcA is subsequently converted into IdoA by Agl32. As such, NMR was performed as in earlier studies that demonstrated Agl32 to be a D-glucuronyl C5-epimerase (Vershinin et al. 2025).

**Figure 1:**
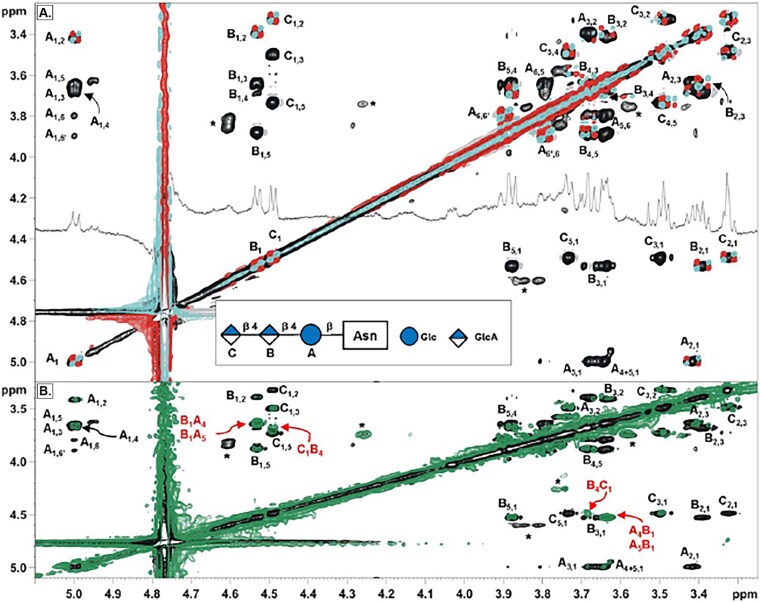
^1^H–^1^H Homonuclear spectra (600 MHz, 298 K, D_2_O) of the *Hbt. *s*alinarum* N-linked glycan from Δ*agl27* strain cells. A) Proton spectrum and overlay of TOCSY (black) and COSY (cyan and red) spectra. The composition of the N-linked trisaccharide is depicted in the inset. B) Overlay of TOCSY (black) and NOESY (green) spectra. The letters used to assign the cross-peaks are as in Table 1. The red letters indicate the inter-residue NOE correlations. “^*^” indicates a density not related to the glycan.

**Figure 2:**
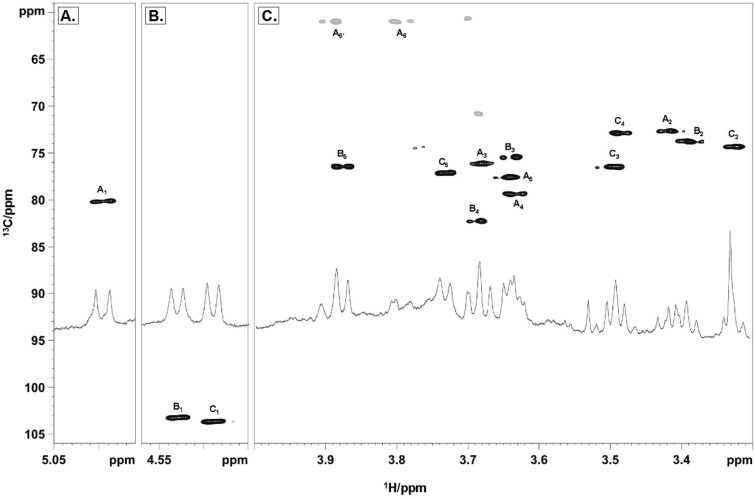
^1^H–^13^C HSQC spectrum (600 MHz, 298 K, D_2_O) of the *Hbt. *s*alinarum* N-linked glycan from Δ*agl27* strain cells. A) The anomeric carbon of the Glc residue (A). B) The anomeric region of the two GlcA units (residues B and C). C) The carbinolic region. The letters used for assigning densities are as in Table 1. “^*^” indicates a density not related to the glycan.

The proton spectrum of the archaellin-linked N-glycan displayed three anomeric signals (~5.0–4.49 ppm) marked with capital letters **A-C** (Fig. 1A) and several high-field signals (3.1–0.7 ppm), likely corresponding to free peptides or to the peptide to which the N-linked glycan is bound (Supplementary Fig. 1). The HSQC spectrum (Fig. 2) revealed an anomeric proton at 4.99 ppm that correlated with a carbon at 80.2 ppm, the value expected for the anomeric carbon atom engaged in an N-glycosidic linkage (Table 1). This signal was labeled **A**_**1**_ and was attributed to a β-Glc unit (^3^*J*_H1,H2_ = 9.4 Hz), as deduced by the efficient magnetization transfer in the TOCSY spectrum that correlated H-1 to all protons of the unit, including the two H-6 s (Fig. 1A). Correlations with the other proton and carbon signals were deduced by analyzing the recorded 2D NMR spectra. The COSY spectrum determined the positions of H-2 (labeled **A**_**2**_) and of H-3 (**A**_**3**_) at 3.41 and 3.68 ppm, respectively (Fig. 1A), while **A**_**4**_ and **A**_**5**_ were found to coincide with **A**_**3**_, in line with the values previously attributed for the N-glycan of the wild-type strain (Notaro et al. 2022). The match in chemical shifts also occurred with the carbon values, indicating that **A** was glycosylated at O-4. Finally, the protons at 3.79 and 3.89 ppm were associated with a hydroxymethyl carbon at 61.0 ppm and were, therefore, labeled as **A**_**6’**_ and **A**_**6**_, respectively (Fig. 1A).

**Table 1:** Proton and carbon (in italics) chemical shifts measured for the glycopeptide isolated from the *Hbt. *s*alinarum* mutant deleted of *agl27* measured at 600 and 150 MHz, respectively, in D_2_O at 298 K.

Residue	1	2	3	4	5	6
**A**	4.99	3.41	3.68	3.63	3.64	3.89;3.79
**4-β-Glc-Asn**	*80.2*	*72.7*	*76.1*	*79.3*	*77.5*	*61.0*
**B**	4.53	3.39	3.64	3.64	3.68	*ND*
**4-β-GlcA**	*103.5*	*73.7*	*75.4*	*82.2*	*76.5*	*ND*
**C**	4.49	3.32	3.49	3.48	3.73	*ND*
**4-β-GlcA**	*103.7*	*74.3*	*76.5*	*72.8*	*77.1*	*ND*

*ND* – not determined

The HSQC spectrum presented two other main anomeric signals at 4.53 and 4.49 ppm, labeled **B** and **C**, respectively. Regarding **B**, the COSY spectrum determined the positions of all protons (Table 1), with the positions of **B**_**3**_ and **B**_**5**_ being confirmed by the corresponding NOE effects (Fig. 1B). This last proton had no other correlations, namely, it missed the protons at position 6 and as such was assigned to a GlcA unit. Analysis of the HSQC spectrum completed the chemical shift assignments, identifying **B** as a GlcA unit, β-configured at its anomeric centers (^3^*J*_H1,H2_ = 8.0 Hz), while the de-shielded value of C-4 (82.2 ppm, Table 1) revealed that **B** was glycosylated at the corresponding position.

The ^3^*J*_H1,H2_ value (7.9 Hz) of the last anomeric signal, labeled **C** (4.49 ppm), indicated the β configuration of the anomeric center, while the COSY spectrum enabled identification of **C**_**2**_ and **C**_**3**_ at 3.32 and 3.49 ppm, respectively. It should be noted that the proton signal at 3.49 ppm correlated with two different carbon atoms in the HSQC spectrum at 76.5 and 72.8 ppm (Fig. 2), indicating that it arose from the overlap of two different carbinolic protons. The NOESY spectrum then allowed identification of the position of **C**_**5**_ at 3.73 ppm, due to its NOE effect with H-1 (Fig. 1B), and since this proton presented a COSY correlation at 3.48 ppm, namely **C**_**4**_, it was clear that this proton was nearly coincident with **C**_**3**_. The assignment of the C-3 and C-4 carbon chemical shifts at 76.5 and 72.8 ppm, respectively, accounted for the reference values reported for GlcA (Speciale et al. 2022). This assignment fits a residue that is not further substituted, namely, a residue located in a terminal position. Any other combination, such as a unit substituted at position 3 or at position 4, would have presented a combination of carbon chemical shift values divergent from those observed in the HSQC spectrum. Finally, H-5 of **C** had no other correlations, characteristic of a GlcA unit.

In terms of the sequence of the sugars, the NOESY spectrum presented a correlation between H-4 of **B** and H-1 of **C** (label **B**_**4**_**C**_**1**_) and between H-4 of **A** and H-1 of **B** (**A**_**4**_**B**_**1**_, Fig. 1B). Based on these considerations, the structure of the glycan was identified by the sequence **C**-**B**-**A**, namely as β-GlcA-(1 → 4)-β-GlcA-(1 → 4)-β-Glc-peptide, as presented in the inset of Fig. 1A. As such, it would seem that conversion of the GlcA initially added to position three of the DolP-bound trisaccharide precursor of the tetrasaccharide into IdoA occurs only after addition of the terminal GlcA. This is reminiscent of what occurs with the eukaryal D-glucuronyl C5-epimerases GlcE and Dsepi1, where only polymer-incorporated GlcA undergoes epimerization, although each enzyme shows a distinct substrate preference in terms of flanking sugars. Whereas GlcE processes GlcA adjacent to N-deacetylated and N-sulfated glucosamine, DSepi1 only needs adjacent GalNAc, although the number of GalNAc-IdoA pairs in the polymer is augmented when dermatan 4-*O*-sulfotransferase 1 (D4ST1) sulfates the GalNAc adjacent to IdoA (Pacheco et al. 2009b; Tykesson et al. 2018). In contrast, Agl32 has much simpler flanking sugar requirements, with only GlcA on either side of the target GlcA being necessary.

NMR also revealed that the GlcA found at the third and final position of the truncated N-linked glycan in *Hbt. salinarum* Δ*agl27* cells is not sulfated. In *Hbt. salinarum*, the complete N-linked tetrasaccharide that decorates glycoproteins is sulfated at the IdoA and GlcA found at glycan positions three and four (Vershinin et al. 2021; Notaro et al. 2022). The findings reported here are thus consistent with earlier mass spectrometry-based results showing that assembly of the complete IdoA-containing tetrasaccharide is a pre-requisite for sulfation (Vershinin et al. 2021; Zaretsky et al. 2025). The earlier observation of the failure of cells lacking Agl32, in which the GlcA at position three of the N-linked tetrasaccharide is not epimerized into IdoA, to sulfate the last two N-linked tetrasaccharide sugars further supports the claim that sulfation follows generation of the IdoA-incorporating tetrasaccharide (Vershinin et al. 2025).

### Structural insight into the workings of Agl32

To date, the three-dimensional structures of three eukaryal D-glucuronyl C5-epimerases have been solved, namely, those of zebrafish GlcE (PDB 4PW2; Qin et al. 2015) and human GlcE (PDB 6HZZ; Debarnot et al. 2019), both responsible for the appearance of IdoA in heparan sulfate, and of human DSepi1 (PDB 6HZN; Hasan et al. 2020), responsible for the appearance of IdoA in dermatan sulfate. In addition, sequence alignment suggests that the only known bacterial D-glucuronyl C5-epimerase (Raedts et al., 2013) relies on a catalytic mechanism similar to that employed by GlcE (Debarnot et al. 2019). Although earlier analysis revealed the human GlcE structure as being significantly different from the predicted structure of Agl32 (Vershinin et al. 2025), the finding reported here that Agl32 requires the target GlcA to be flanked by GlcA on either side called for a closer comparison of the predicted Agl32 structure with those of the three solved D-glucuronyl C5-epimerase structures.

**Figure 3:**
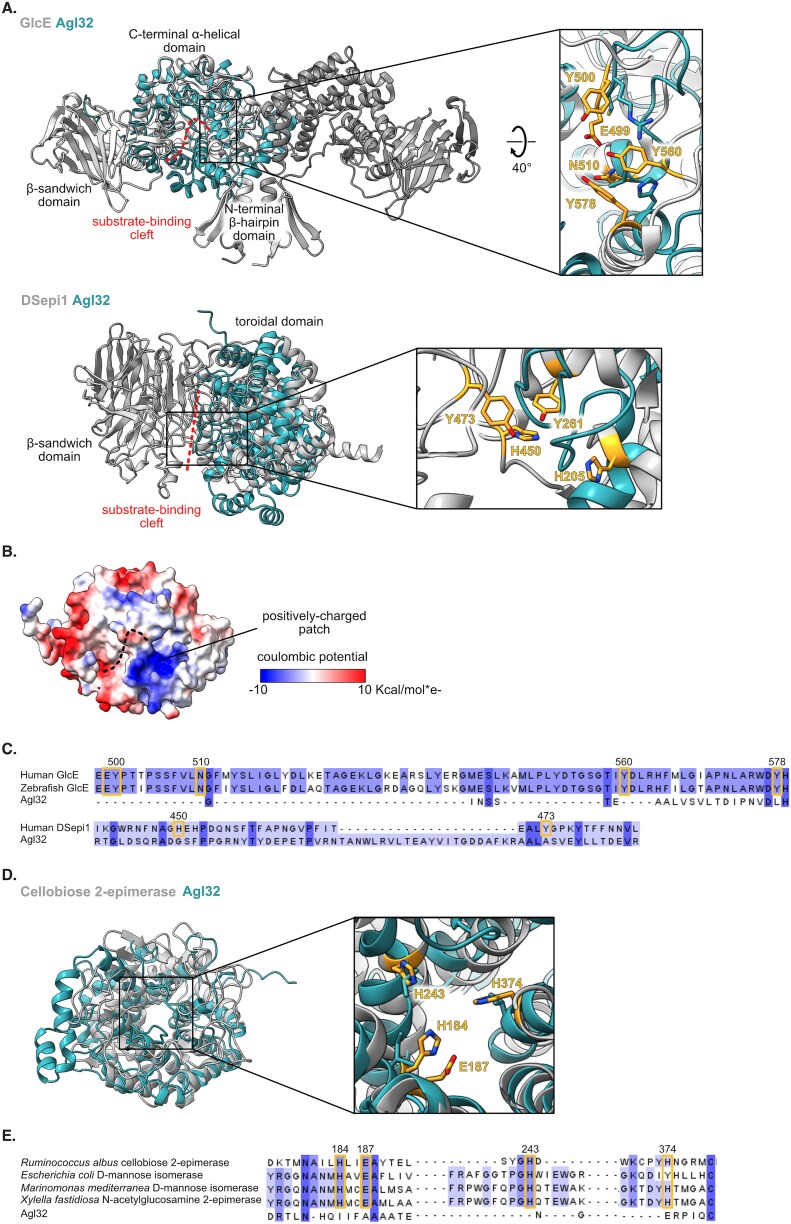
Structural comparison of Agl32 with eukaryotic and bacterial epimerases. A) Super-positioning of the AlphaFold-predicted model of Agl32 (teal) and human GlcE (top, grey; PDB 6HZZ) or human DSepi1 (bottom, grey; PDB 6HZN), showing that Agl32 contains only the α-helical domain and lacks the additional domains present in GlcE and DSepi1. Substrate-binding clefts of GlcE and Dsepi1 are indicated by red dashed lines. Right – Close-up views of the GlcE and DSepi1 active sites, with catalytic residues colored orange. In those regions where the main chain of Agl32 overlaps with that of the human enzymes, Agl32 residues are shown as sticks. B) Electrostatic surface map of the predicted Agl32 model, with that region corresponding to the substrate-binding cleft in GlcE indicated by a black dashed line. C) Multiple sequence alignment of the active site regions of human and zebrafish GlcE (top; residue numbering according to the human GlcE sequence) and human DSepi1 (bottom), colored according to the degree of conservation (purple, conserved; white, variable). Catalytic residues are indicated by orange boxes. D) Super-positioning of *Ruminococcus albus* cellobiose 2-epimerase (grey; PDB 3VW5) and the predicted model of Agl32 (teal). Right – Close-up view of the active site, with *R. albus* cellobiose 2-epimerase catalytic residues colored orange. Equivalent spatial positions in Agl32 are shown as sticks. E) Multiple sequence alignment of the active site regions of those bacterial epimerases identified as best matching the predicted Agl32 structure in a Foldseek search. Coloring reflects the degree of conservation (purple, conserved; white, variable). Catalytic residues are indicated by orange boxes. Residue numbering follows that of *R. albus* cellobiose 2-epimerase sequence.

Human and zebrafish GlcE adopt a conserved dimeric architecture, in which each monomer comprises three domains, namely, an N-terminal β-hairpin domain that mediates dimerization, a central β-sandwich domain, and a C-terminal α-helical domain that harbors the catalytic site (Qin et al. 2015; Debarnot et al. 2019). In contrast, human DSepi1 contains a central toroidal domain enriched in α-helices and loops, along with a β-sandwich domain, but lacks the β-hairpin domain found in GlcE, consistent with its monomeric state (Hasan et al. 2020). As Agl32 is predicted to consist solely of an α-helical domain, lacking the β-hairpin domain required for GlcE dimerization, it is thus likely to be monomeric. At the same time, Agl32 exhibits only partial structural alignment with the helical domains of both GlcE and DSepi1, with structural super-positioning yielding root-mean-square deviation (RMSD) values of 5.0 Å over 184 residues for human GlcE (PDB 6HZZ), 4.3 Å over 184 residues for zebrafish GlcE (PDB 4PXQ), and 6.1 Å over 232 residues for human DSepi1 (PDB 6HZN), indicating limited structural similarity (Fig. 3A). The predicted model of Agl32, colored according to probability, is presented as Supplementary Fig. 2.

Substrate-binding considerations were next considered. The substrates of GlcE and DSepi1 are accommodated within extended, positively charged clefts in these enzymes. Specifically, GlcE acts on substrates at least six sugars-long, relying on a binding cleft found within the α-helical domain that accommodates approximately seven sugars (Fig. 3A, top; Debarnot et al. 2019). Similarly, DSepi1 activity requires a substrate comprising a minimum of five sugars, with its substrate-binding cleft, located between the toroidal and β-sandwich domains (Fig. 3A, bottom), being capable of accommodating eight sugars (Hasan et al. 2020). The substrates processed by GlcE and Dsepi1 are thus longer than the tetrasaccharide processed by Agl32. Predicted to contain a single α-helical domain and lacking an extended cleft of comparable size to that seen in the eukaryal D-glucuronyl C5-epimerases, Agl32 would appear to rely on a distinct mode of substrate binding. Indeed, super-positioning of Agl32 with human GlcE suggests that several Agl32 loops would sterically clash with the canonical GlcE binding groove. Instead, an adjacent positively charged surface patch in Agl32 could serve as an alternative binding interface (Fig. 3B), consistent with the shorter substrate requirement of Agl32, which acts on the third sugar of a tetrasaccharide and thus calling for only a limited binding surface.

The conservation of catalytic residues was also addressed. In GlcE, the key catalytic residues are E499, Y500, N510, Y560, and Y578 (numbering according to the human enzyme; Debarnot et al. 2019). None of these residues are present at corresponding spatial positions in the predicted Agl32 model, indicating a distinct active-site architecture and catalytic mechanism. Likewise, the catalytic residues of DSepi1, namely, H205, Y261, H450, and Y473 (Pacheco et al. 2009c; Hasan et al. 2020), are not conserved in Agl32 and indeed are located in a region that does not overlap with the Agl32 model. Moreover, sequence alignment does not reveal conservation of catalytic residues between Agl32 and either GlcE or DSepi1 (Fig. 3C). It would thus appear that Agl32 relies on an active site, and hence, mechanism, distinct from that of known D-glucuronyl C5-epimerases.

Given the lack of similarity to eukaryotic D-glucuronyl C5-epimerases, an unbiased structural search was performed by comparing the predicted Agl32 model against structurally related proteins identified using Foldseek (van Kempen et al. 2024). This search revealed that the closest structural homologues of Agl32 are a bacterial cellobiose 2-epimerase (see Vershinin et al. 2025), D-mannose isomerase, and N-acetylglucosamine 2-epimerase, all members of the N-acylglucosamine 2-epimerase (AGE) super-family (Saburi et al. 2018). These enzymes are all monomeric proteins comprising a single α-helical domain (Itoh et al. 2008; Fujiwara et al. 2013; Saburi et al. 2018), which exhibits partial structural overlap with the α-helical domain of Agl32. Specifically, overlapping the Agl32 structure with that of the cellobiose 2-epimerase (PDB 3VW5; Fujiwara et al. 2013) yielded an RMSD value of 5.3 Å over 296 residues. The catalytic residues of the bacterial enzyme, namely, H184, E187, H243, and H374 (Fujiwara et al. 2013), are not conserved in the predicted Agl32 structure (Fig. 3D). Sequence alignment did not reveal conservation of active site residues with those of the other AGE super-family members either (Fig. 3E), indicating that Agl32 is unlikely to share the same active site architecture or catalytic mechanism as these enzymes, despite their overall structural similarity.

### Gene organization analysis supports the involvement of Agl32 homologues in N-glycosylation by other haloarchaea

Earlier phylogenetic analysis involving a BLAST search using *Hbt. salinarum* Agl32 as query not only revealed the existence of Agl32 homologues in other archaea but also distinguished these sequences from homologues of the only other archaeal sugar epimerase described to date, i.e. *Haloferax volcanii* AglQ (Arbiv et al. 2013; Notaro et al. 2022). However, because nothing is presently known about N-glycosylation in those Agl32 homologue-containing species, no further insight into whether these homologues serve a role similar to that served by *Hbt. salinarum* Agl32, namely, as a D-glucuronyl C5-epimerase, was offered.

**Figure 4:**
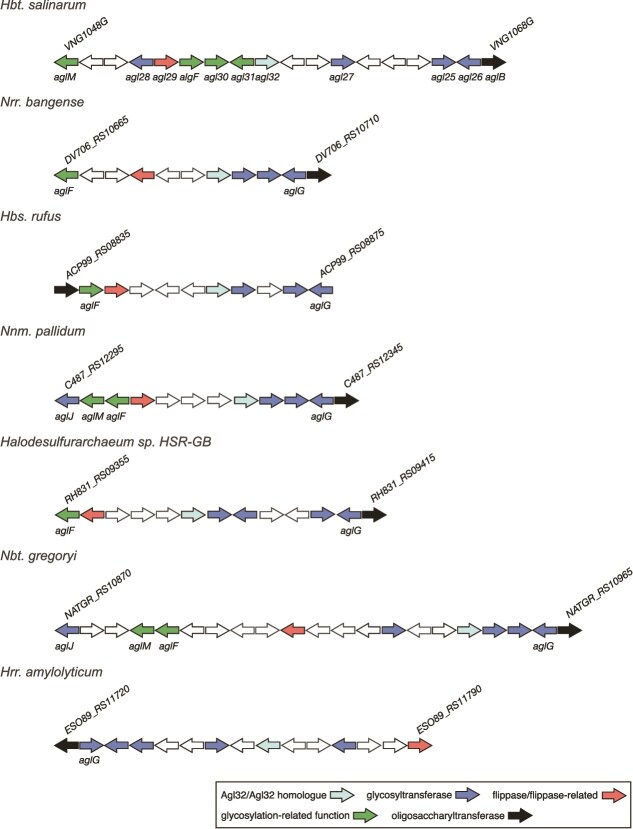
Mapping of putative N-glycosylation genes in haloarchaea predicted to encode Agl32 homologues. Gene islands anchored by putative *aglB* sequences encoding the archaeal oligosaccharyltransferase that also include other putative N-glycosylation pathway-encoding genes, including Agl32 homologues, in various haloarchaeal species are presented. The color-coded putative function of a given gene product is defined in the legend. Where a given gene has been annotated as encoding a known archaeal N-glycosylation pathway component, the gene is named accordingly. In addition, the locus tags of the first and last genes in each island are indicated.

Archaeal N-glycosylation-related genes can be organized into clusters anchored by *aglB*, encoding the archaeal oligosaccharyltransferase, a key component of N-glycosylation pathways (Magidovich and Eichler 2009; Kaminski et al. 2013; Nikolayev et al. 2020). Indeed, this is the case for genes encoding proteins comprising the *Hbt. salinarum* Agl pathway used for N-glycosylation (Zaretsky et al. 2019; Vershinin et al. 2021; Vershinin et al. 2023; Vershinin et al. 2025; Zaretsky et al. 2025)(Fig. 4). As such, finding that Agl32 homologue-encoding genes exist in similar *aglB*-anchored gene clusters would support the assignment of these homologues as also being D-glucuronyl C5-epimerases. Accordingly, genes adjacent to those encoding Agl32 homologues in *Halobellus rufus, Halodesulfurarchaeum* sp. HSR-GB, *Halorubrum amylolyticum, Natrinema pallidum, Natronobacterium gregoryi* and *Natronorubrum bangense* (encoding protein products sharing 46%-54% identity with *Hbt. salinarum* Agl32, with 97%-99% query coverage) were addressed. In each of these strains, the Agl32 homologue-encoding sequence was found in a gene island that not only encoded a sequence annotated as encoding an oligosaccharyltransferase but also sequences annotated as encoding glycosyltransferases, including those previously shown to participate in archaeal N-glycosylation (i.e. AglG (Yurist-Doutsch et al. 2008) and AglJ (Kaminski et al. 2010), flippases or auxilliary N-glycosylation pathway components (i.e. AglF, a glucose-1-phosphate uridyltransferase, and AglM, a UDP-glucose dehydrogenase (Yurist-Doutsch et al. 2010))(Fig. 4). These observations thus argue that the participation of the D-glucuronyl C5-epimerase Agl32 in N-glycosylation, and hence, the incorporation of IdoA into an N-linked glycan, is not unique to *Hbt. salinarum* but rather may occur in other haloarchaea. However, confirming the presence of IdoA in N-linked glycans generated by these species, as well as the contribution of Agl32 homologues, awaits the development of appropriate molecular tools for working with these species.

Finally, a multiple sequence alignment of *Hbt. salinarum* Agl32 and its haloarchaeal homologues from the species listed above was performed in the hope of gaining insight into the catalytic workings of the archaeal D-glucuronyl C5-epimerase. Such analysis revealed that while the seven sequences considered are quite conserved, there are few histidine, glutamate, or tyrosine residues in the regions corresponding to the active site regions of the other epimerases considered in this study (Supplementary Fig. 3).

## Conclusions

*Hbt. salinarum* N-glycosylation presents aspects of this universal post-translational modification seemingly unique to these archaea, such as the transient methylation of the glycan at the DolP-bound level, sulfation of IdoA at the O-3 position and, indeed, the incorporation of IdoA in the N-linked tetrasaccharide (Eichler et al. 2026). Having recently identified Agl32 as the D-glucuronyl C5-epimerase responsible for the appearance of this IdoA (Notaro et al. 2022), the current study considered how this archaeal enzyme compared with its better described eukaryal counterparts involved in GAG biosynthesis. Specifically, aspects of the workings of Agl32, relative to human GlcE and DSepi1, were addressed. NMR spectroscopy confirmed that like GlcE and DSepi1, Agl32 also requires the target GlcA to be bordered by sugars on either side. On the other hand, structure-based approaches revealed that Agl32 can be clearly distinguished from GlcE and DSepi1 in terms of enzyme structure, the mode of substrate binding and catalytic mechanism. Finally, the seemingly simple substrate requirements of the smaller archaeal enzyme, specifically a requirement for GlcA on either side of the target sugar as part of a short polysaccharide, rather than more complex sugars found as part of a longer polymer as required by GlcE and DSepi1, raise the possibility of using Agl32 to generate IdoA for biotechnological and medical applications.

## Materials and methods

### Cell growth

*Hbt. salinarum* NRC-1 (ATCC strain 700922) parent strain cells were grown in medium containing 250 g NaCl, 20 g MgSO_4_·7H_2_O, 3 g sodium citrate, 2 g KCl, 10 g peptone per l, supplemented with 50 μg/mL uracil at 42 °C, as were Δ*agl27* strain cells (Darnell et al. 2017; Vershinin et al. 2021). The absence of *agl27* in the deletion strain was confirmed by quantitative PCR, as previously described (Vershinin et al. 2021; Zaretsky et al. 2025).

### Isolation of archaella

*Hbt. salinarum* archaella were isolated from the spent growth medium (30 l) of Δ*agl27* strain cultures as previously described (Alam and Oesterhelt 1984). Briefly, cultures were grown to logarithmic phase (OD_600_ ~ 0.8) and left for 24 h at room temperature without shaking. The cultures were centrifuged for 30 min (6000× g, 15 °C). The supernatant (post-spin 1 supernatant) was collected and centrifuged again for 15 min (16,000× g, 15 °C). The supernatant (post-spin 2 supernatant) was removed and the pelleted material was resuspended by shaking in 1 mL of 4 M basal salt solution (250 g NaCl, 20 g MgSO_4_·7H_2_O, 3 g sodium citrate, 2 g KCl per l) and heated for 10 min at 90 °C. The heated suspension was centrifuged for 15 min (16,000× g, 15 °C). The resulting supernatant (post-spin 3 supernatant) was maintained at 4 °C for 24 h and centrifuged for 2 h (40,000× g, 4 °C). After removal of the supernatant (post-spin 4 supernatant), the pellet (post-spin 4 pellet) was lyophilized and stored at −20 °C until further analysis.

### Isolation of an N-linked trisaccharide-containing glycopeptide

Isolation of N-linked glycan-containing peptides from archaellins comprising the enriched archaella was performed as previously reported (Notaro et al. 2022; Vershinin et al. 2025). Briefly, the lyophilized archaella-containing samples (140 mg) were resuspended in 3 mL of H_2_O and digested with proteinase K (1 mg/mL). The resulting N-glycosylated peptides were separated from other peptide fragments by size-exclusion chromatography using a Biogel P2 column (total volume 20 mL, flow rate 12 mL/h, DDW as eluant). The eluate was monitored with a refractive index detector, pooled accordingly, and analyzed by proton NMR to identify those fractions enriched in glycopeptides. Additional purification was achieved by anion exchange chromatography (Q-Sepharose fast-flow; Cytiva, Buccinasco, Italy; total volume 0.5 mL). Elution was performed using NaCl solutions of increasing ionic strength (10 mM, 100 mM, 200 mM, 400 mM, 700 mM, 1000 mM; three volumes of each), followed by a final wash with 1 M NaOH, immediately neutralized to pH 7 after collection. Eluates with the same ionic strength were pooled and desalted on a Biogel P2 column (same conditions as above). This additional purification step efficiently removed traces of starch, as well as other peptides that previously co-eluted with the glycopeptide of interest. The N-linked trisaccharide-containing peptide (6 mg) eluted at higher ionic strength (100 mM NaCl).

### NMR analysis

N-linked trisaccharide-containing peptide samples were dissolved in deuterated water (550 μL), and spectra were recorded on a Bruker 600 DRX apparatus equipped with a CryoProbe at 298 K. Homonuclear ^1^H–^1^H 2D experiments (COSY, TOCSY, NOESY) were recorded using 512 FIDs of 2048 complex points with 48 scans per FID. Mixing times of 100 and 500 ms were used for TOCSY and NOESY spectra, respectively. ^1^H–^13^C HSQC spectrum was acquired with 512 FIDs of 2048 complex points by accumulating 80 scans. All spectra were calibrated on internal acetone (^1^H 2.225 ppm, ^13^C 31.35 ppm). Transformation and analysis of the spectra were performed using Bruker Topspin 4.3.0 software.

### Structural comparisons

The structure of Agl32 was predicted using AlphaFold (Jumper et al. 2021). RMSD values between the predicted model of Agl32 and experimental structures of glucuronyl C5-epimerase epimerases (PDB 4PXQ, 6HZZ, 6HZN and 3VW5) were calculated using the cealign function in PyMol. Foldseek (van Kempen et al. 2024) was used to search the PDB for structures most similar to the predicted Agl32 model. Multiple sequence alignment was performed using Clustal Omega (Sievers et al. 2011) and visualized in Jalview (Waterhouse et al. 2009). Molecular graphics figures were prepared using UCSF ChimeraX (Pettersen et al. 2004), with surface electrostatic potential being calculated using the “coulombic” command.

## Supplementary Material

Supplementary_Materials_rev_cwag045

## Data Availability

Date reported in this study are available from the corresponding author upon reasonable request.
