# Supplementary material for: Distinct properties of Halobacterium salinarum Agl32, an archaeal D-glucuronyl C5-epimerase involved in N-glycosylation
Source: Glycobiology. 2026 Jun 11;36(8):cwag045. doi: 10.1093/glycob/cwag045 (PMC13308652; doi:10.1093/glycob/cwag045)
Supplement: Supplementary_Materials_rev_cwag045 [file supplementary_materials_rev_cwag045.docx]

**Supplementary Materials**

**Distinct properties of *Hbt. salinarum* Agl32, an archaeal D-glucuronyl** **C5-epimerase involved in N-glycosylation**

Antonella Aquilone^1^, Yarin Levi^2^, Marianna Zaretsky^2^, Zlata Vershinin^2^, Iris Grossman-Haham^2,3^, Cristina de Castro^1^ and Jerry Eichler^2,*^

^1^Department of Chemical Sciences, University of Napoli Federico II, Naples, Italy

^2^Department of Life Sciences, Ben-Gurion University of the Negev, Beersheva, Israel

^3^The Ilse Katz Institute for Nanoscale Science and Technology, Ben-Gurion University of the Negev, Beersheva, Israel

*Correspondence and material requests should be sent to: Prof. Jerry Eichler, Dept. of Life Sciences, Ben Gurion University of the Negev, P.O. Box 653, Beersheva 84105, Israel; Tel: +972 8646 1343; Email: jeichler@bgu.ac.il


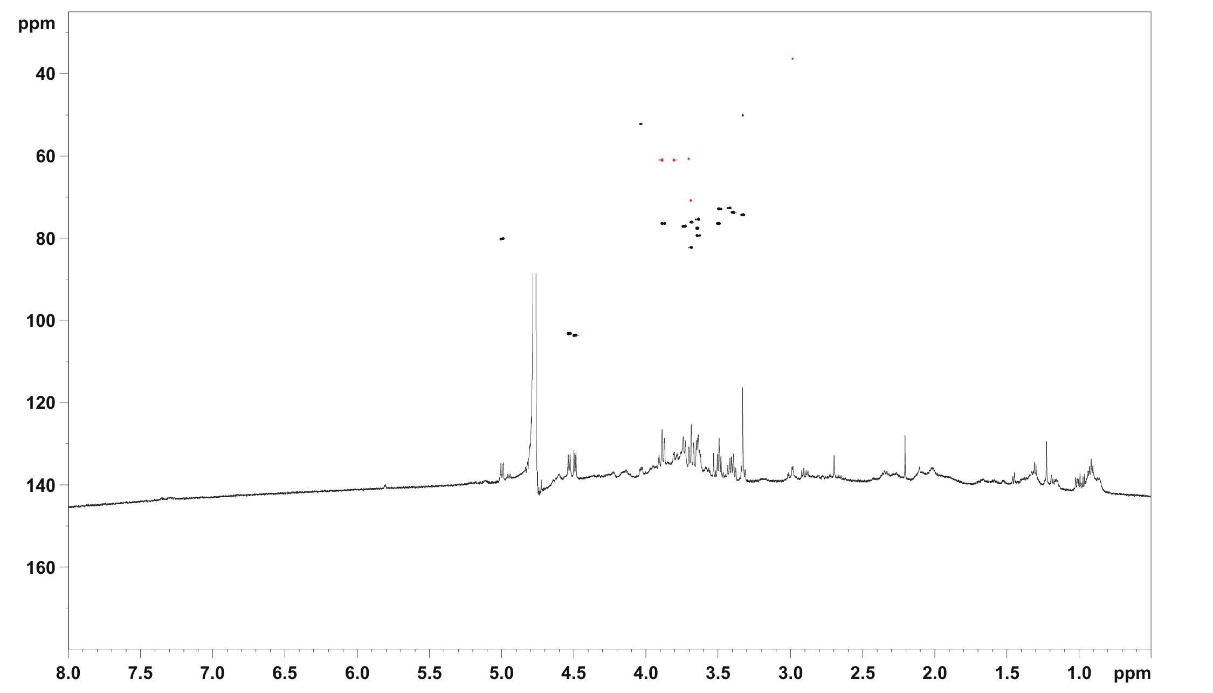


**Supplementary Fig. 1 -** Full-range HSQC spectrum measured for the N-glycosylated peptide isolated from the *Hbt. salinarum* cells deleted of *agl27* (600 MHz, 298 K).

**
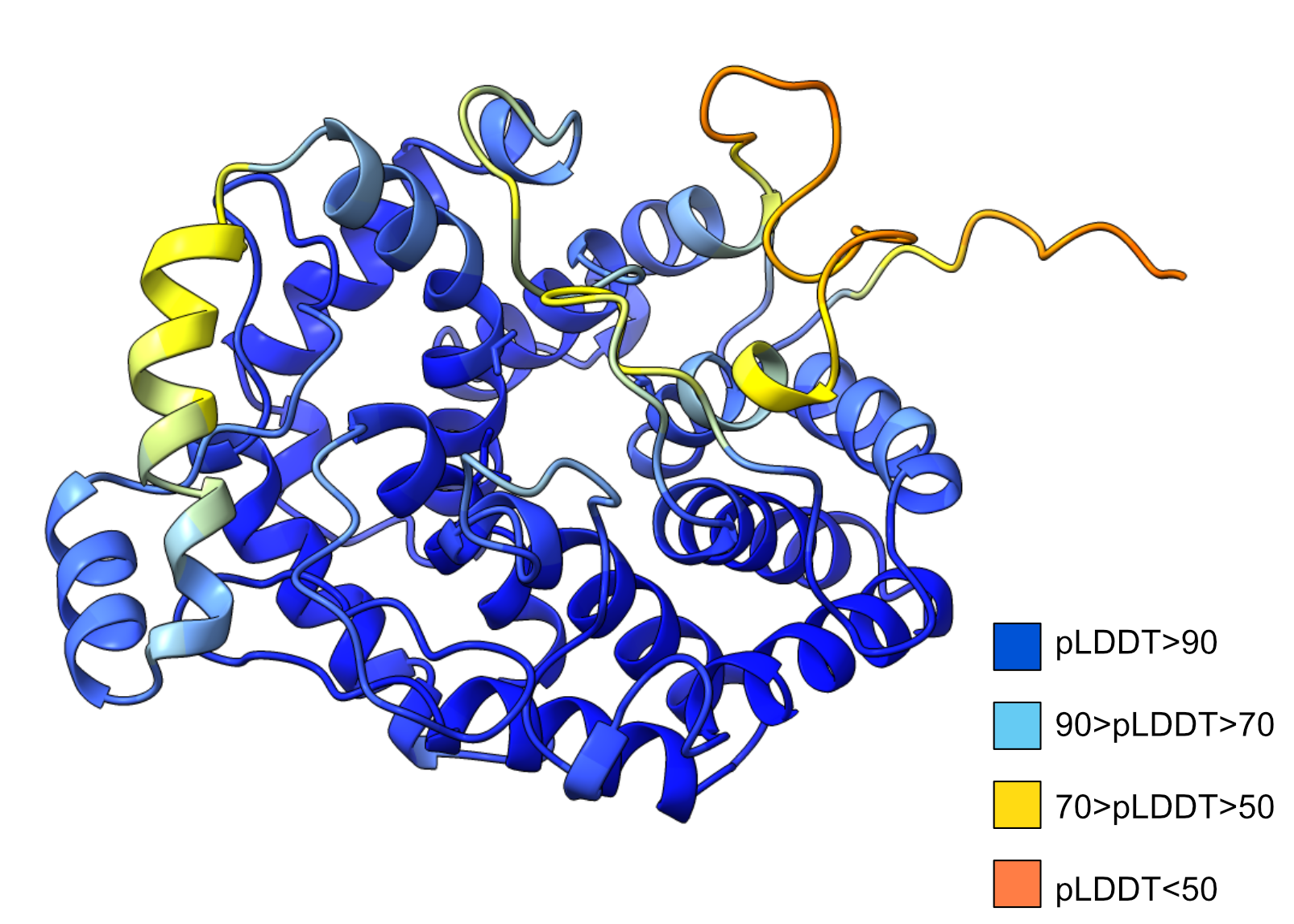
**

**Supplementary Fig. 2 -** The AlphaFold-generated model of Agl32, colored according to probability.

**Supplementary Fig. 3 –** Multiple sequence alignment of putative haloarchaeal Agl32 homologues. Positions containing identical residues are indicated by asterisks, conserved residues by colons and semi-conserved residues by periods. The sequences compared were *Hbt. salinarum* Agl32, *Halobellus rufus* WP_081927313, *Halodesulfurarchaeum* sp. HSR-GB WP_310553926.1, *Halorubrum amylolyticum* WP_128905688, *Natrinema pallidum* WP_006186104, *Natronobacterium gregoryi* WP_005579330.1 and *Natronorubrum bangense* WP_006064614.
